# 

**Published:** 1875-05

**Authors:** 


### CONTENTS:

### ORIGINAL COMMUNICATIONS.

ARTICLE I.—Notes on Celluloid. By Prof. James B. Hodgkin, D. D. S. .......... 1

ARTICLE II.—Building on a Part of the Crowns of Teeth. By E. L. Hunter, D. D. S. .......... 4

ARTICLE III.—Letter from Dr. T. B. Gunning .......... 5

ARTICLE IV.—Letter from * * * .......... 6

Susquehanna State Dental Association .......... 7

Illinois State Dental Association .......... 7

Kentucky State Dental Association .......... 8

### SELECTED ARTICLES.

ARTICLE V.—Gold. By G. V. Black, D. D. S. .......... 8

ARTICLE VI.—The Eye Tooth and the Eye. By Dr. Jas. Campbell .......... 17

ARTICLE VII.—Artificial Teeth on Protruding Gums. By Dr. W. E. Driscoll .......... 21

ARTICLE VIII.—Treatment of Burns and Scalds. By Jno. Morris, M. D. .......... 23

ARTICLE IX.—Lancing Gums. By Charles E. Buckingham, M. D. .......... 26

ARTICLE X.—Aconite as a Local Anæsthetic By Paul W. Allen, M. D. .......... 26

ARTICLE XI.—The Relation of Imperfect Teeth to Cataract. .......... 31

ARTICLE XII.—Local Treatment of Cancer of the Mouth. By J. E. Nichols, M. D. .......... 33

ARTICLE XIII.—A Fundamental Requisite .......... 35

ARTICLE XIV.—A New Method of Applying the Rubber Dam. By S. F. Ham, M. D. .......... 37

ARTICLE XV.—New Base for Teeth .......... 38

### EDITORIAL, ETC.

The Thirty-Sixth Annual Commencement of the Baltimore College of Dental Surgery .......... 39
The Right to Use Celluloid .......... 42

### BIBLIOGRAPHICAL

Transactions of the New York Odontological Society .......... 43
The Chemists' and Druggists' Diary for 1875 .......... 43
A Protest .......... 43
Reflex Irritations Throughout the Genito-Urinary Tract .......... 44
A Case of Reflex Neuralgia .......... 44
Fifth Report of the New York Ophthalmic and Aural Institute .......... 44
The Sanitarian .......... 44
The Pen and Plow .......... 44
The Herald of Health .......... 44

### MONTHLY SUMMARY.

Death from Chloroform .......... 45

On Right-Handedness .......... 45

Detection of Falsification of Wax .......... 46

Death from Methylene .......... 46

Poisoning by Carbolic Acid .......... 47

Teeth and Oatmeal .......... 47

Relief of Pain by External Use of Chloral .......... 48

Oleate of Mercury in Treatment of Ringworm .......... 48

An Indigestible Morsel .......... 48

